# Healthy Recreations

**Published:** 1857-12

**Authors:** 


### HEALTHY RECREATIONS.

AMUSEMENT is as much a necessity to the mind as food is to the body. The mind is vivified by pleasurable recreations as much as the body is sustained by a nutritious diet. But not less transient and deceptive as the aids which opium, and tobacco, and alcohol afford the body, are novel-reading and theatrical performances the unsubstantial quickeners of the mind and heart. And as nothing gives the body more enduring strength than plain substantial meat and bread, so the intellect and the affections are strengthened by the exercise of those real benevolences which every-day life, in cities especially, so loudly call for.

The dollar spent for a seat in the theatre amuses its occupant for a few short hours, and after they are past, there is nothing real to look back upon. That same dollar spent upon one of the thousands of the children of want in any large community, would make that poor child feel rich for a day, and would lift up and happify his stricken heart, as often as remembered, for many long days to come; while, as to the donor's, it will be a sweet thing to think of, even in a dying hour. Let our recreations then be, not in sham and show, but in sweet realities.

